# Spatial correlation between malaria cases and water-bodies in *Anopheles sinensis* dominated areas of Huang-Huai plain, China

**DOI:** 10.1186/1756-3305-5-106

**Published:** 2012-05-31

**Authors:** Shui-sen Zhou, Shao-sen Zhang, Jian-jun Wang, Xiang Zheng, Fang Huang, Wei-dong Li, Xian Xu, Hong-wei Zhang

**Affiliations:** 1 National Institute of Parasitic Diseases, Chinese Center for Disease Control and Prevention; WHO Collaborating Centre for Malaria, Schistosomiasis and Filariasis; Laboratory of Parasite and Vector Biology, Ministry of Health, No.207 Rui Jin Er Road, Shanghai, People’s Republic of China; 2 Anhui Province Center for Disease Control and Prevention, No.12560 Fanhua Road in Economic-Technological Development Zone, Hefei, People’s Republic of China; 3 Henan Province Center for Disease Control and Prevention, Eastern Nongye Road, Zhengzhou, People’s Republic of China

## Abstract

**Background:**

Malaria re-emerged in the Huang-Huai Plain of central China during 2006–2008, dominated with *Anopheles sinensis* as a vector. However, there is no information on strategies based on multi-factor analysis to effectively control the re-emergence of malaria in these areas. Previous experience indicates some relationship between the distribution of water bodies and malaria cases, but more detailed data are not available and in-depth studies have not been conducted up to now. The objective of this study was to identify the relationship between the distribution of water bodies and presentation of malaria cases using spatial analysis tools in order to provide guidance to help formulate effective strategies for use in controlling the sources of malaria infection, based on the identification of risk areas and population.

**Methods:**

The geographic information of malaria cases and their surrounding water bodies were collected from Suixi, Guoyang, Guzhen, Yingshang, Fengyang and Yongqiao County in Anhui province, Yongcheng and Tongbai County in Henan province. All malaria cases distributed in 113 villages in these 8 counties were collected from the China Information System for Disease Control and Prevention and confirmed by household investigation. Data on GIS and malaria cases were mapped and analyzed with the software of ArcGIS 9.2 to identify the spatial correlation between malaria cases and water bodies. The distance from households with malaria cases to the nearest water bodies was used to calculate the *OR* value by *Chi-square* test. The risk area was identified through the comparison of *OR* values in different distances.

**Results:**

357 malaria cases and their GPS data as well as surrounding water bodies were collected and analyzed. 74% of malaria cases were located within the extent of 60 m proximity to the water bodies. The risk rate of people living there and presenting with malaria was significantly higher than others (*OR* = 1.6,95%*CI* (1.042, 2.463),*P* < 0.05).

**Conclusions:**

The results revealed that distribution of water bodies is an important factor influencing the occurrence and distribution of malaria cases in the *An.sinensis* areas, and implies that the scope and population within 60 m around water bodies are at risk and could be a targeted population for case management of malaria.

## Background

Historically the Huang-Huai Plain of central China was seriously epidemic for Malaria, and the number of cases in these areas was up to 21.99 million, accounting for 91.2% of the total reported cases in the country in 1970. With active implementation of malaria control measures for more than 30 years, considerable success had been achieved and the cases decreased dramatically and many counties have reached the standard of the basic malaria elimination (the incidence was less than 1/10000) [1]. But from early in the 21st century, malaria re-emerged in the central parts, especially the Anhui and Henan Provinces in Huang-Huai Plain located along the latitude 33°N with an average yearly temperature of 16 °C. Total 64,178 malaria cases and 52,082 suspected cases with 38 deaths were reported in 917 counties of 23 Provinces in 2006, most malaria cases in this year with 66.4% coming from Huang-Huai Plain [2]. Afterwards, the re-emergence was effectively controlled under the enhancive control strategies and the support from the Global Funds, but the number of malaria cases and the incidence in this area has still contributed to more than 60% of the total cases in the country in recent years [3].

The main malaria vectors in this area are *An. sinensis* and *An. anthropophagus* in the past, but in recent years the density of *An. anthropophagus* was very low, and the re-emergence is mainly caused by *An. sinensis* which is developed either in the streams and pools or accumulated water on the ground [4]. These mosquitoes are less sensitive to vector controls such as indoor residual spraying (IRS) and insecticide-treated bed-nets (ITNs) because this species is campestral and its sucking habit is on cattle and pigs. Thus the control of infection sources is the major malaria control strategy in these areas [4,5]. Therefore, it is necessary to define the extent of infection sources for case management such as case detection, foci identification and anti-relapse treatment. The malaria cases are the major source of infection and the distribution of malaria cases matches well with the location of mosquito larval habitats [6-9] which is the leading vector in this area, inhabiting small water bodies (paddy fields, gullys, *etc.*) [10]. As there is limited information about the spatial characteristics of water bodies and their relationship to the distribution of malaria cases in China, the objective of this study was to explore the spatial relationship between water bodies and malaria cases.

## Methods

### Study sites and subjects

**Figure 1:**
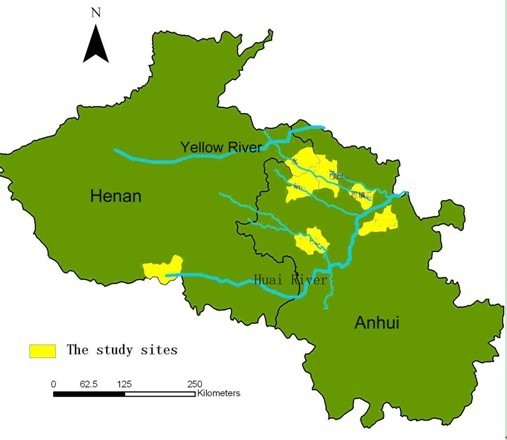
The map shows the location of the study sites and the Huang-Huai plain.

According to the identification of the position (upper\middle\lower of the region) and the diversity of topographical features, 113 villages (Anhui 90, Henan 23) in 8 counties (6 counties in Anhui: Fengyang, Guzhen, Suixi, Guoyang, Yingshang, Yongqiao County; 2 counties Henan: Yongcheng County and Tongbai County) were selected by stratified random unequal proportion sampling methods. All the counties were located at latitude 32°17′ ~ 34°18′ N, longitude 113° ~ 117°09′E along the Yellow River and Huai River, with malaria re-emergence in recent years (Figure 1). The information of malaria cases in these counties were obtained from the National Malaria report system in 2004 ~ 2007, confirmed by household investigation.

### Data collection

1. The data collection of water bodies

**Figure 2:**
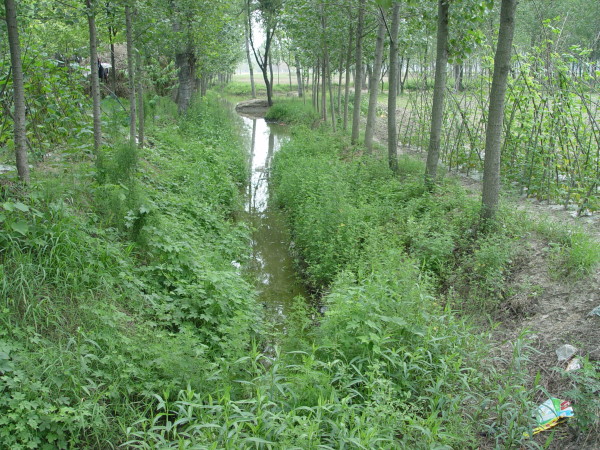
** Water bodies. a.** Shows the stream around the villages, which are considered and defined to be one kind of water body.** b**. Shows another kind of water body: accumulated water on the ground in the villages.

The term “water bodies” refers to the paddy fields, gully and little stream, which are suitable breeding sites of *An. sinensis* (Figure 2a, b). The identification of water bodies was conducted under the supervision of experienced experts who had been working on vector surveys for more than 20 years. The data was collected during the season that was suitable for malaria transmission, which is from June to October. At that time, it is easy to define the breeding sites of *An. sinensis* by finding the larvae in the water. The data collection of malaria cases and the location of households in the village.

Based on reported malaria cases in the target villages household investigations were conducted to confirm all the epidemiological information and especially to make sure there were no invalid cases. The geographic information on all the residences including malaria cases and water bodies in the villages were collected using a hand-held GPS (Global Positioning System, GPS) locator.

### Data analysis

1. Database development: The software of Mapsource was used to transform GPS data into Excel format for establishing the database.

2. Spatial analysis: The distances between houses of malaria cases and water bodies were calculated with software ArcGIS 9.2. Buffer analysis was conducted based on multiple buffer rings with water bodies to be the center. The Straight—Line Distance Function was used to calculate the distances from households of cases to the nearest water bodies, based on the results of buffer analysis.

3. Statistical analysis:

3.1 The straight-line distances from the houses of cases to the nearest water bodies were analyzed using software SPSS 11.5. The descriptive statistical analysis was conducted to get the statistical distribution of distance from house to water bodies and get the general characters of these data.

3.2 Based on the results of 3.1, all the residents in each village were grouped according to the characteristics of the distribution of malaria cases, and chi-square test was used to analyze and evaluate the risk of malaria transmission.

## Results

**Figure 3:**
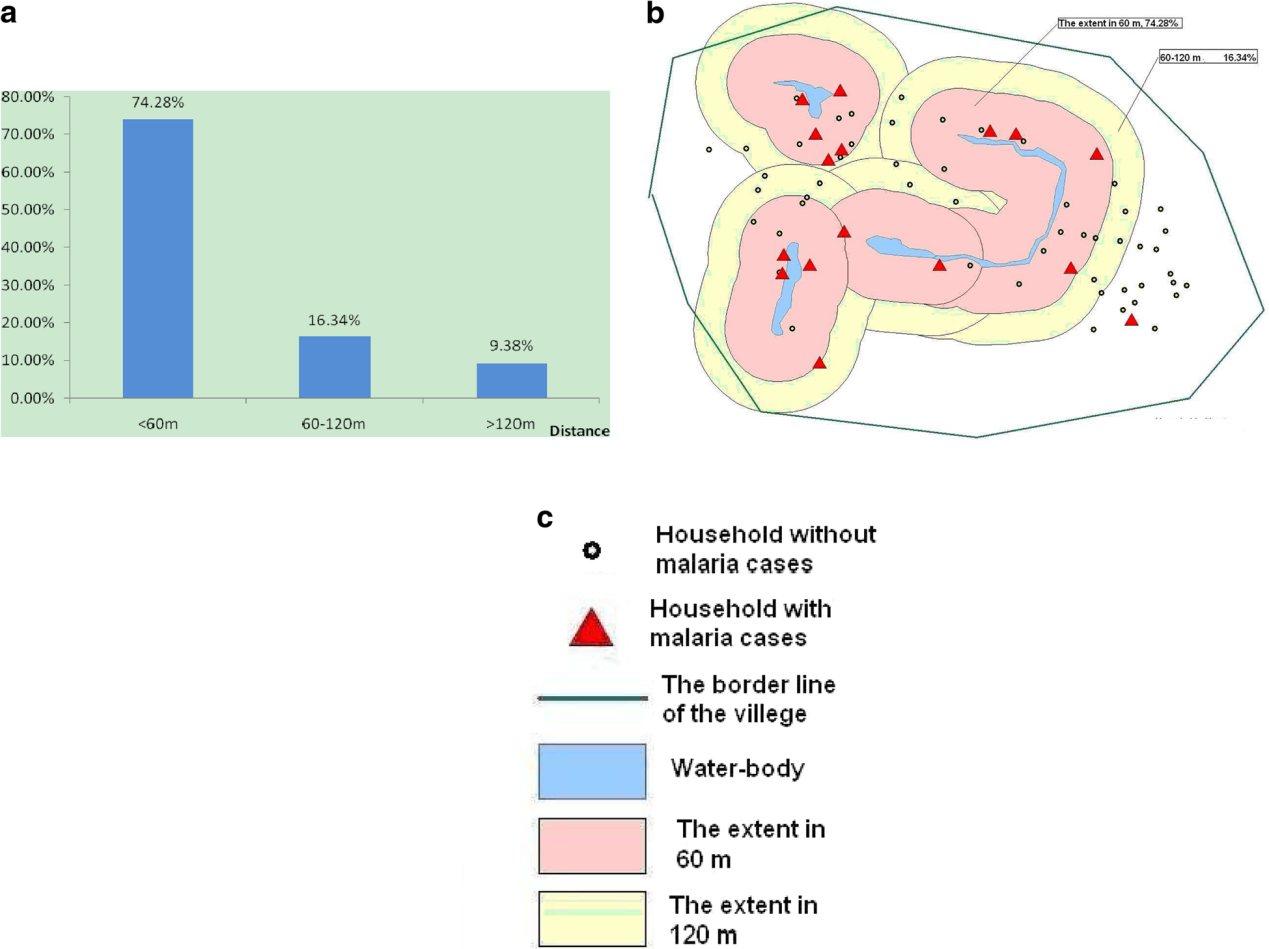
** a. The percentage of malaria cases at different distances from the water bodies. b.** The general spatial characteristics of malaria cases distributed in the villages with three groups (see legend).

**Figure 4:**
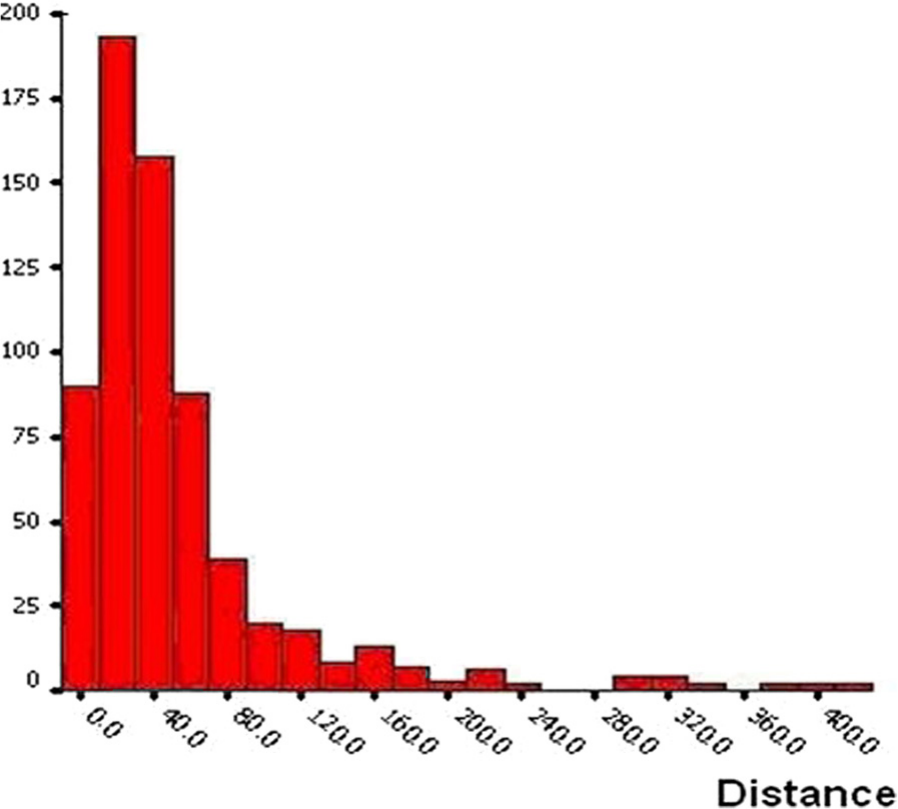
The statistical distribution of distance from house to water bodies.

1. The spatial correlation between malaria cases and water bodies of 246 households with malaria cases and more than 300 water bodies were collected from geographic data by GPS in the 90 villages from 6 counties in Anhui. The descriptive statistic results show that the distances from households of cases to the nearest water bodies had a positive-skew distribution (Figure 3), the median was 60.9 m. 74.28% (182/246) of cases were distributed at a distance ≤ 60 m, 16.34% (40/246) of them were located at a distance of 60-120 m, and 9.38% (182/246) were scattered over 120 m. (Figure 4).

2. The risk area of malaria transmission

**Figure 5:**
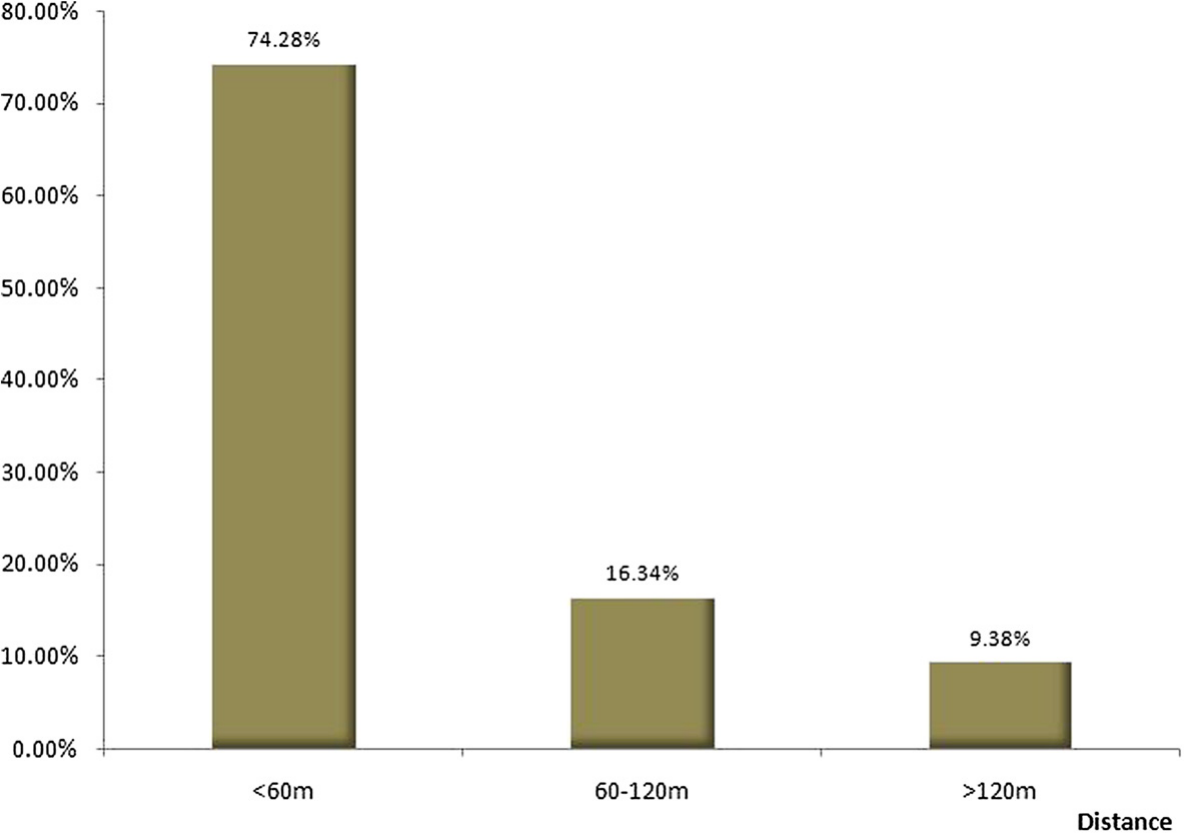
The percentage of malaria cases at different distances from water bodies (study sites in Anhui).

**Figure 6:**
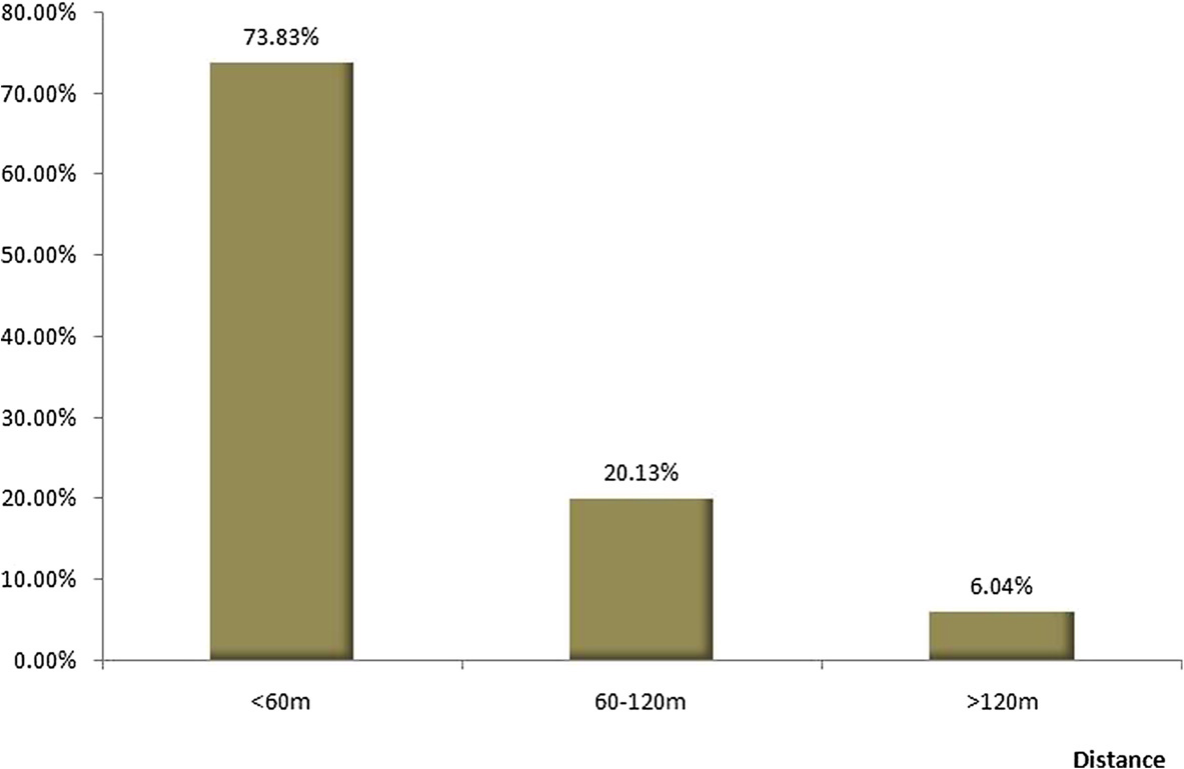
The percentage of malaria cases at different distances from water bodies (study sites in Henan).

The data from 587 households and 141 confirmed cases as well as information on water bodies collected in 7 villages in Yongcheng County and Tongbai County were tested, and compared to make sure there was consistency between studies in Henan and Anhui (showed in Figure 5 and Figure 6).

These data of residents were grouped according to different distances from their houses to the nearest water bodies. Based on the spatial correlation between malaria cases and water bodies, locations of residents were classified into different groups in every 5 m distance. A significant difference is seen only in the 60 m group (*P*<0.05) (Table 1). The *χ*2 test was conducted to analyze the difference between proportions of case and non-case (Table 2).

**Table 1:** **The* Chi-square *and* OR *value of Different group division**

**Group**	**chi-square**	***P* value**	***OR***	**95%CI**	
50 m	0.919	0.338	1.16	0.851	1.593
55 m	3.125	0.077	1.332	0.964	1.84
60 m	4.664	0.041	1.602	1.042	2.463
65 m	3.398	2.306	2.706	1.869	3.918

The Table 1 showed Chi-square and OR value of Different group division and presented that how the cut-off 60 m was identified.

**Table 2:** Comparison of the risk between two groups

**Group**	**No. of households**		**Total**
	**No-cases**	**Cases**	
≦60 m	290	101	391
> 60 m	161	35	196
Total	451	136	587

The Table 2 showed the *χ*2 analysis of two groups (no-cases and cases).

It is implied that 60 m may be the critical division of malaria risk areas. Grouping the residents in each village by the distance from their houses to the nearest water bodies (> 60 m and ≤60 m), the total number of households in group ≦60 m was 391(290 without malaria cases and 101 with malaria cases) while in the group > 60 m, there are 196 households with 35 malaria cases. The proportion of malaria cases in the group ≦60 m is significantly higher than that of the group > 60 m (*χ*2 = 4.664,*P* < 0.05; *OR* = 1.602, 95%CI (1.042,2.463), *P* < 0.05). It concluded that people living within the extent of 60 m near water bodies have more risk of malaria infection.

## Discussion

As a vector-borne disease, malaria transmission has a close relationship with the biological and natural factors due to the vectors—anopheles. Currently *An. sinensis* is the sole or major vector in the Huang-Huai Plain, this mosquito is campestral and its blood feeding habit is on cattle and pigs, and its larval habitats are the small water bodies such as ponds, paddy fields, gullies, and so on [11,12]. Vector control measures such as ITN are not effective in these areas. Thus case management is critically important especially in the interventions to risk population around mosquito breeding -water bodies. The previous studies [9,13,14] found a negative relationship between the risk of malaria infection and the distance from the anopheles breeding sites to the houses: the closer residents live to anopheles breeding sites, the higher the risk of malaria infection becomes, due to the higher density of anopheles. Entomological survey found that the anopheles prefer to lay eggs near the blood meal sites, which results in an increase of vector density, thus causing the increase of malaria risk among people living close to larval habitats. However, little information about the relationship between larval habitat distribution and malaria cases can be obtained in this area in the historical literature. We evaluated the relationship between malaria prevalence and the distribution of water bodies, and obtained similar results to the previous studies on other anopheles spp. [15-21].

All of the study sites belong to under-developed counties and have similar social factors, such as the cultivation (the main crops are wheat, rice, corn, soybean, *etc.*), architectural style of houses (only windows in the front of house), and daily habits (most of the residents prefer to sleep outside in summer). So, excluding the effect of social factors, the results of our study indicated that the increase of malaria risk near the water bodies could be attributed to the anopheles habitat.

The Huang-Huai Plain has been malaria endemic many times, according to the previous national reports. The main strategies used for control was anti-malaria treatment, given to all the people or administration of the medicine to all the people for prevention [10]. However, as these strategies were expensive and had low compliance, they were abandoned in some counties. Some suggestions based on our results have been given for rolling back re-emergence and controlling transmission of malaria, this includes: the high risk population should be identified based on the distance from housing to water bodies (≤60 m) which is more cost effective; People living in the risk area are the targeted population who should take some preventative treatment; mosquito breeding sites around housing needs to be cleared, taking measures particularly against larvae.

## Conclusion

The Huang-Huai Plain was malaria endemic area, according to the previous national reports. The main strategies used for control was anti-malaria treatment, given to all the people or administration of the medicine to all the people for prevention [10]. This study combined investigation in the field and spatial analysis on computer, to describe the character of malaria cases distribution in the village level and develop the risk map. The results revealed that distribution of water bodies is an important factor influencing the occurrence and distribution of malaria cases in the *An.sinensis* areas, and implies that the scope and population within 60 m around water bodies are at risk and could be a targeted population for case management of malaria.

## Competing interests

The authors declare that they have no competing interests.

## Authors’ contributions

SSZ was responsible for the overall study and involved in all stages of this study including design, field work, data analysis and writing the manuscript. SSZ was the focal point in this study and involved in field work and data analysis and wrote the manuscript. JJW, XZ, FH, WDW, XX and HWZ performed the collection of malaria incidence data in study sites. All authors read and approved the final manuscript.

## References

[B1] Tang LH Progress in malaria control in China Chin Med J 2000 115 69 92 11775219

[B2] Zhou SS Wang Y Tang LH Malaria situation in the People’s Republic of China in 2006 Chin J Parasitol and Parasit Dis 2007 25 6 439 411 18441886

[B3] Zhou SS Wang Y Xia ZG Malaria situation in the People’s Republic of China in 2009 Chin J Parasitol and Parasit Dis 2011 29 1 1 3 21823314

[B4] Ministry of Health Disease Prevention and Control Bureau Handbook for malaria control and prevention 2007 People's Hygine Publishing House, Beijing 98 99

[B5] Pan B The morphological characteristics, ecological habit, malaria transmission effect of malaria vectors in China J Trop Med 2003 3 4 477 480

[B6] Allen WH Maurice O Richard O Richard O Aggrey JO Altaf AL Bernard LN William AH A Geographic Information System applied to a malaria field study in western Kenya Am J Trop Med Hyg 1998 58 3 266 272 9546401 10.4269/ajtmh.1998.58.266

[B7] Foley D Torres E Mueller I Bryan JH Bell D Host-dependent Anopheles flavirostris larval distribution reinforces the risk of malaria near water Trans Roy Soc Trop Med Hyg 2003 97 283 287 10.1016/S0035-9203(03)90143-X 15228242

[B8] Noboru M Pamela S Guiyun Y Influence of host and larval habitat distribution on the abundance of African malaria vectors in western Kenya AmJTrop Med Hyg 2002 67 1 32 38 10.4269/ajtmh.2002.67.32 12363061

[B9] Thomas C Lindsay S Local-scale variation in malaria infection amongst rural Gambian children estimated by satellite remote sensing Trans R Soc Trop Med Hyg 2000 94 159 163 10.1016/S0035-9203(00)90257-8 10897355

[B10] Zhou ZJ Study of malaria control and prevention in China 1991 People's Hygine Publishing House, Beijing 98 117

[B11] Gou GX Li DF Shang LY Guo XS Wang WX Sun QL The study on ecological habits of Anopheles sinensis in Guantang, Luyi County from 1971 to 1996 Chin J Vector Biol & Control 1998 9 2 133 134

[B12] Hu YX Miao YG Fan TB The further study on ecological habits of Anopheles sinensis in the area along Yellow River and Huai River Chin J Parasitol Parasit Dis 1988 S1 135

[B13] Foley D Torres E Mueller I Stream-bank shade and larval distribution of the Philippine malaria vector Anopheles flavirostris Med Vet Entomol 2002 16 347 335 10.1046/j.1365-2915.2002.00382.x 12510886

[B14] Jean-Francois T Lefebvre-zante E Fabrice L Gora N Hilaire B Pierre D Gerard S Vector density gradients and the epidemiology of urban malaria in Dakar, Senegal AmJTrop Med Hyg 1992 47 2 181 189 10.4269/ajtmh.1992.47.181 1354414

[B15] Noboru M Clifford M John I John CB Guiyun Y Spatial distribution and habitat characterization of Anopheline mosquito larvae in western Kenya Am J Trop Med Hyg 1999 61 6 1010 1016 10674687 10.4269/ajtmh.1999.61.1010

[B16] Richard C Kamini N Donald R Spatial targeting of interventions against malaria Bull World Health Org 2000 78 12 1401 1411 11196487 PMC2560653

[B17] Gunawardena D Wickremasinghe A Muthuwatta L Weerasingha S Rajakaruna J Senanayaka T Kotta PK Attanayake N Carter R Mendis KN Malaria risk factors in an endemic region of Sri Lanka, and the impact and cost implications of risk factor-based interventions Am J Trop Med Hyg 1998 58 5 533 542 9598437 10.4269/ajtmh.1998.58.533

[B18] Michael TW Jamie TG Thomas SC Neil MF Maria-Gloria B Azra CG Modelling the impact of vector control interventions on Anopheles gambiae population dynamics Parasit Vectors 2011 4 153 10.1186/1756-3305-4-153 21798055 PMC3158753

[B19] Eliningaya JK Zhou GF Thomas MG Yaw A Mramba N Andrew KG Guiyun Y Predation efficiency of Anopheles gambiae larvae by aquatic predators in western Kenya highlands Parasit Vectors 2011 4 128 10.1186/1756-3305-4-128 21729269 PMC3141748

[B20] Louis CG Jean-Sébastien D Romain G Sebastien B Guy L Didier F Spatial and temporal distribution patterns of Anopheles arabiensis breeding sites in La Reunion Island - multi-year trend analysis of historical records from 1996–2009 Parasit Vectors 2011 4 121 10.1186/1756-3305-4-121 21708013 PMC3145585

[B21] Marianne ES Michael JB Sylvie M Theeraphap C Anand PP William HT Peter WG Iqbal RFE Caroline WK Ralph EH Simon IH The dominant Anopheles vectors of human malaria in the Asia-Pacific region: occurrence data, distribution maps and bionomic précis Parasit Vectors 2011 4 89 10.1186/1756-3305-4-89 21612587 PMC3127851

